# What is the role of community capabilities for maternal health? An exploration of community capabilities as determinants to institutional deliveries in Bangladesh, India, and Uganda

**DOI:** 10.1186/s12913-016-1861-0

**Published:** 2016-11-15

**Authors:** Ligia Paina, Lalitha Vadrevu, S. M. Manzoor Ahmed Hanifi, Joseph Akuze, Rachel Rieder, Kitty S. Chan, David H. Peters

**Affiliations:** 1 Department of International Health, Johns Hopkins Bloomberg School of Public Health, 615 N. Wolfe Street, Baltimore, MD 21205 USA; 2 Indian Institute of Health Management Research, 1 Prabhu Dayal Marg, Sanganer, Jaipur, 302029 India; 3 Health System and Population Studies Division, icddr,b, GPO Box 128, Dhaka, 1000 Bangladesh; 4 Department of Health Policy, Planning, and Management, Center of Excellence for Maternal and Newborn Health Research, Makerere University School of Public Health, New Mulago Hospital Complex, Kampala, Uganda; 5 DKT International Tanzania, Meranani St, Dar es Salaam, Tanzania; 6 Department of Health Policy and Management, Johns Hopkins Bloomberg School of Public Health, 624 North Broadway, #633, Baltimore, MD 21205 USA

## Abstract

**Background:**

While community capabilities are recognized as important factors in developing resilient health systems and communities, appropriate metrics for these have not yet been developed. Furthermore, the role of community capabilities on access to maternal health services has been underexplored. In this paper, we summarize the development of a community capability score based on the Future Health System (FHS) project’s experience in Bangladesh, India, and Uganda, and, examine the role of community capabilities as determinants of institutional delivery in these three contexts.

**Methods:**

We developed a community capability score using a pooled dataset containing cross-sectional household survey data from Bangladesh, India, and Uganda. Our main outcome of interest was whether the woman delivered in an institution. Our predictor variables included the community capability score, as well as a series of previously identified determinants of maternal health. We calculate both population-averaged effects (using GEE logistic regression), as well as sub-national level effects (using a mixed effects model).

**Results:**

Our final sample for analysis included 2775 women, of which 1238 were from Bangladesh, 1199 from India, and 338 from Uganda. We found that individual-level determinants of institutional deliveries, such as maternal education, parity, and ante-natal care access were significant in our analysis and had a strong impact on a woman’s odds of delivering in an institution. We also found that, in addition to individual-level determinants, greater community capability was significantly associated with higher odds of institutional delivery. For every additional capability, the odds of institutional delivery would increase by up to almost 6 %.

**Conclusion:**

Individual-level characteristics are strong determinants of whether a woman delivered in an institution. However, we found that community capability also plays an important role, and should be taken into account when designing programs and interventions to support institutional deliveries. Consideration of individual factors and the capabilities of the communities in which people live would contribute to the vision of supporting people-centered approaches to health.

**Electronic supplementary material:**

The online version of this article (doi:10.1186/s12913-016-1861-0) contains supplementary material, which is available to authorized users.

## Background

Advances in health have not yet reached all those who are in need and, where there has been progress, the benefits have been unequally spread across populations. This is particularly true for maternal health, where access to safe deliveries for the poor is among the most unequal of primary care services across regions of the world [1]. While the world has witnessed a significant fall in maternal mortality in the past three decades, the maternal mortality ratio in developing countries is 14 times higher than in developed ones [2]. Furthermore, only half of all women in developing countries receive the recommended levels of health services [2]. Through the Sustainable Development Goals, “ensur[ing] healthy lives and promot[ing] well-being for all at all ages” [2] remains an important global priority, and integrated, people-centered approaches lie at the core of the vision for supporting resilient health systems and communities [3]. The World Health Organization has drafted a strategy on integrated people-centered health services that recognizes the importance of empowering and engaging individuals, families, and communities in the co-production of care, as well as in voicing their needs [3]. The strategy also identifies gaps in the current knowledge around how to measure progress towards the establishment of people-centered systems, including the role of communities [3]. A better understanding of what community capabilities are, including how to enhance community engagement to draw upon those capabilities and to improve peoples’ health would serve to narrow these knowledge gaps and contribute towards improvements in maternal health.

In our research, communities are defined as “groups of people having common interests, resources, beliefs, needs, occupations or other social conditions that characterize the identity of members and affect their cohesiveness,” with the recognition that this definition must be adapted to a particular geographical and political context [4, 5]. Communities have both individual and collective capabilities [4]. Individual level capabilities involve people’s separate “material, social, and psychological assets” [4, 6], while community capabilities are multi-dimensional and dynamic concepts that involve the collective capabilities of the community. Community level capabilities include social capital (i.e. the resources based on the relationships and networks within a community), as well as various collective physical, human and social resources [4, 5, 7, 8]. Community capabilities empower communities to identify, mobilize and address social problems in a community, as well as to foster and transfer the skills, knowledge, systems and resources that affect community member’s lives [4, 5, 7–9]. Community empowerment is the aspect of community capabilities that involves the process of mobilization and expansion of the capability of individuals and groups to participate in, negotiate with, influence, control, and hold accountable institutions that affect their lives [10, 11].

In order to better understand how to empower communities, the research conducted through the Future Health Systems (FHS) research consortium examines “how communities can be active participants in the planning, delivery, monitoring, and evaluation of their health system, by identifying individual and collective capabilities in social, political, and institutional environments” [5]. Since 2012, FHS has sought to develop approaches to refine the definition of community capabilities and to measure them in various contexts, as a first step towards a vision of supporting change in community capabilities, as well as being able to measure this change over time. Whereas there is wide recognition of the importance of community capability and the related concepts of social capital, community empowerment, or community competence [7, 12–15], there is considerable frustration described in the literature on developing appropriate metrics [10]. It is difficult to develop standardized, comparable measures of community capabilities when such capabilities are often highly contextualized.

The concepts of capability (including the more specific concepts of empowerment and social capital) is multidimensional and highly variable [9, 11]. The Shortened Adapted Social Capital Assessment Tool (SASCAT) was developed in 2005 and used to assess social capital among individuals living in Peru, Vietnam, India and Ethiopia [16]. Another instrument to measure community empowerment is the Community Capabilities Index (CCI), which was developed in Sudan to measure community empowerment as related to natural resource governance. While illustrative of the potential applicability of such community empowerment indices, the CCI does not address capabilities as they relate to health [17]. Lippman and colleagues recently developed an instrument to assess community mobilization in the context of HIV programming [18] that includes 7 domains. In Zambia, Underwood and colleagues measured and validated community capacity through 6 community-generated domains, to assess the roles of these domains in the context of health communication interventions focused on developing community capacity [19]. The Zambian example represents one of the few experiences to date that also examined the association between community capacity and community action for health [19]. An important challenge is to develop valid and reliable measures of community capabilities. Besides a few exceptions [19], very few reliable and valid measures of community capabilities exist, particularly for low income settings. Furthermore, the link between community capabilities and health outcomes or access to health services, such as those for maternal health services has seldom been explored. It may be that the effects of community capabilities differ according to the outcome of interest. The objectives of this paper is first to describe the development of a community capability score based on the FHS project’s experience in Bangladesh, India, and Uganda, and, second, to empirically examine the role of community capabilities as determinants of institutional delivery in these three contexts.

### Country context

In 2012, the FHS teams in Bangladesh, India, and Uganda undertook the implementation of a common household survey to test whether and how community capability can be measured. Each of the teams worked in a unique context, and focused on different research questions. The team in Bangladesh, for example, aimed to work with village doctors to improve their integration with the formal health care system and improve the overall quality of care received by women and children in rural and remote areas. The India team worked in the Sundarbans, a fragile, disaster-prone environment, with an intention to enhance the knowledge of communities, NGOs, and service providers about health and barriers to access, in order to better provide quality and timely care. The Uganda team worked across several rural districts to implement a multi-pronged intervention aimed at reducing barriers to institutional delivery and post-natal complications for pregnant women.

All of the country teams struggled with a common contextual problem: mothers living in study areas faced many barriers to access to quality maternal and neonatal health services. In all three countries, maternal mortality remained very high: 176 per 100,000 live births in Bangladesh; 174 per 100,000 live births in India, and 343 per 100,000 live births in Uganda [20]. A closer review of these statistics at the sub-national level revealed a direr situation. For example, in Chakaria Upazila of Bangladesh, 80 % of the deliveries happened at home [21], the majority probably not attended by a skilled birth attendant. Institutional deliveries in Chakaria remained low, particularly for mothers in the lowest income quintile, where only 8 % of the poorest women delivered in a health facility, compared to 40 % of richest ones [21]. In the Sundarbans region in West Bengal, India, maternal mortality was a bit lower than the national average (141 per 100,000 live births), but the majority of maternal deaths happened at home [22]. Although women attended ante-natal care visits, the vast majority would not return to the facility to deliver and probably very few had access to skilled birth attendants when delivering at home [23]. In Uganda, the evaluation conducted by the FHS team revealed that, within the rural areas where their study was conducted, around 75 % of women delivered in a health facility where the intervention was implemented, compared to 65 % of women in control areas [24].

## Methods

The FHS project research teams have begun implementing their interventions in 2011. The teams began their work under the cross-cutting “Unlocking Community Capabilities” theme by developing approaches to measure community capability, in order to understand social relations and resources within and across the communities they worked in [5]. In each setting, a baseline, cross-sectional household questionnaire was conducted in 2012, to which the teams added a module of questions to assess the presence or absence of key community capability. Each of the survey components are explained in greater detail below.

*Sampling and study area description*: For this article, we pooled survey data collected from Bangladesh, India, and Uganda.

In Bangladesh, all of the data was collected from the Chakaria Health and Demographic Surveillance System (CHDSS). The CHDSS includes 20,036 households that are eligible to participate in the survey. The UCC questionnaires and the household data were collected from the same sample, but at two different points in time. For the UCC survey, the team selected only one household member from each household, randomly, using a sampling fraction of 400/population of the age group with the lowest population size, for men and women separately. The total number of individuals selected was 5152 (2188 men and 2964 women). Data on safe motherhood practices was collected from mothers of infants through three monthly household visits, as part of the CHDSS [25]. The data from the CHDSS and the UCC questionnaire were combined and matched by household ID. The final sample was comprised of 1238 women of reproductive age (15–49 years old), whose last child was born with the past 5 years.

In India, data on community capability, maternal and child health and demographic questions were collected as part of the baseline survey. The survey included 1200 households in both deltaic and non-deltaic regions of the Patharpratima Block in West Bengal, India. The sample was selected using probability proportionate to size (PPS), where the sampling unit was the village (equivalent to a community). Thirty out of 87 villages were selected. Within each village, households were selected at random, based on whether any children under 5 lived in a particular household. For the purpose of this analysis, the data on institutional deliveries reflects the information provided by mothers about their most recent birth. The final sample consisted of 1199 women of reproductive age (15–49 years old).

In Uganda, data on the community capability were collected as a cross-sectional study annexed to the household baseline survey for MAternal and NEwborn care practices STudy (MANEST) among women who had delivered within 1 year prior the survey from 3 districts. For the community capability data, 369 household heads (both men and women) out of 2011 households selected for the MANEST survey were selected across 3 districts in the North-Eastern Part of Uganda. One parish from each of the 17 sub-counties that make up the 3 districts were randomly selected and 369 household heads out of the 2011 households were randomly selected to complete the community capability questionnaire. The data from the community capability questionnaire was linked to the MANEST data using a unique household identifier in order to obtain the institutional delivery (main outcome) and the individual level predictor variables used in this analysis.

In all countries, the sample was restricted to women of reproductive age (15–49 years old), whose last child was born in the past 5 years.

*Outcome variable*: The outcome of interest in this paper was “institutional delivery”, whether or not a woman delivered in an institution. In the survey, women were asked “Whether or not a woman delivered in an institution” (Bangladesh), “Where was the child born” (India), or “Where did you deliver from” (Uganda). The delivery counted as an institutional delivery if the child was born at a hospital, regardless of ownership by government or non-government sector. The delivery was not counted as institutional if the child was born at home or elsewhere (e.g. with a traditional birth attendant or en route to the hospital or health facility).

### Community level predictor variables

The FHS team developed a series of quantitative community capability questions based on a thorough literature review conducted at the beginning of the project (see Additional file 1). These questions were intended to be used across countries, in household surveys, exit surveys, or any other quantitative surveys, though they could also be used in mixed methods research. The community capability questions developed by the FHS team spanned several conceptual domains: community/village assets (inclusive of 9–13 services, such as schools, that were offered in a community), community organizations (examining both the general existence of organizations, as well as the extent to which community members participate and/or benefit), civic voice actions (e.g. voting in a local election), community coherence and decision-making (e.g. about commitment to collective goals), and health system problems (e.g. absence of doctors). Because these concepts are multi-dimensional and highly contextualized, each of the country teams had the flexibility to select the domains and questions that were most relevant to their research projects. For the analysis presented here, we selected to use only the domains and questions that were common across all of the three countries, in order to ensure that the same level of details was available. The common domains specifically considered for this analysis include: community/village assets (the physical and organizational resources of a community to which the community members should have access, and the ability of communities to mobilize resources for collective use), group participation (the community capacity to engage its members in collective action, and the degree to which members are active in group functions), and community cohesion (the forces that act on members of a community to retain and actively contribute to the community or the degree to which members want to be part of a group and are loyal and united in pursuit of group goals). A total of 13 common community capability variables were identified and are listed in Table 1. While the original survey recorded respondents’ answers on a 5-point Likert scale (except in India, where community coherence was measured on a 4-point Likert scale), for the purposes of the analysis reported here, we dichotomized answers (i.e. 0 = if Likert scale denoted poor structures or disagreement (including here the mid-range value of the scale) and 1 = if Likert scale denoted fair or better structures or agreement).

**Table 1:** Summary of common community capability variables

Common community capability items	Details
Community/Village assets	Schools
	Water supply
	Electricity/solar system
	Roads
	Health facilities
	Television
	Radio
Community coherence	Collective Goals: As members of this community we are all committed to the same collective goals
	Develop solutions: I am confident that we as community members can develop and carry out solutions to problems as they arise
	Different views: People with differing views are able to equally contribute their views on community plans and activities.
	Different economic status: People from different economic status in this community are able to equally contribute their views on community plans.
	Women contributions: Women in this community are able to equally contribute their views on community plans and activities.
Group participation	Membership in any community organization

*Individual level predictor variables*: For this analysis, we extracted information that was found in all three data sets, such as maternal age, education, parity, and antenatal care participation. In the pooled dataset, maternal age was classified as: 15–24 years old, 25–34 years old, and 35–49 years old. Maternal education was categorized as no formal education; primary education; lower secondary education; higher secondary education; and university or higher. Parity was categorized as one child, two to four children, and 5 or more children. Antenatal care participation was classified as: none; one or two visits; and three or more visits.

*Statistical analyses*: All analyses were performed using Stata 14 [26]. In the first phase, we used exploratory factor analysis to identify the key factors behind community capability across the three country setting. We selected factors if they had eigenvalues exceeding 1.0, verifying the percent variance explained, cumulative percent of variance explained, and their scree plots [27]. Our main factor analysis was based on polychoric correlations, an analysis which is best suited for data that are not normally distributed, and we used varimax rotation to facilitate interpretation of identified factors. Three factors had eigenvalues >1, with and the loadings for each variable are summarized in Table 2. Internal consistency reliability was measured with the Cronbach’s alpha coefficient, for which a value of 0.7 is considered acceptable [28].

**Table 2:** Factor loadings for community capabilities items from the pooled dataset

Common community capability items	Variable	Factor 1	Factor 2	Factor 3	Unexplained
Community assets	Schools	0.0051	−0.0227	0.0317	0.0143
	Water supply	−0.0035	−0.0689	0.0283	0.0544
	Electricity/solar system	−0.0100	0.9150	0.0215	0.0509
	Roads	0.0014	−0.0224	0.0195	0.0085
	Health facilities	−0.0335	0.1274	−0.1954	0.1143
	Television	0.0710	0.3636	−0.0066	0.1706
	Radio	−0.0187	−0.0750	−0.0128	0.0575
Community coherence	Collective goals	0.1054	−0.0379	0.4343	0.1443
	Develop solutions	−0.0321	0.0301	0.8697	0.0749
	Different views	0.6932	−0.0063	0.0584	0.1571
	Different economic status	0.7074	−0.0065	−0.0920	0.1474
	Women contributions	0.0130	−0.0044	0.0462	0.0266
Group participation	Membership in any community organization	0.0050	−0.0235	−0.0012	0.0119
	Summated scale’s Cronbach’s alpha	0.68			

The Cronbach’s alpha coefficient was 0.68, suggesting that the reliability of our scale was marginally acceptable. The pooled data showed that the community capability variables grouped into logically cohered latent components. Because all community capability variables represented essential aspects of community capability and for ease of interpretation, we developed a simple summative and unweighted community capability index score. We generated at the individual respondent level as the number of items declared per respondent out of all the 13 possible items. Given the limited number of items, we chose a single community capability score rather than three separate scores. The score we obtained was then translated into percentages, so that the final variable was a continuous variable from 0 to 100. We tried to aggregate this score at the community level, commonly identified across the surveys as the village level. However, this was not possible due to very small sample sizes.

In the second phase of the analysis, we combined the community capability score with the household survey data. Individual-level determinants of institutional deliveries included maternal age, education, parity, number of visits to ante-natal care, and country of origin. The community-level determinant of institutional deliveries was the community capability score.

We produced descriptive summary statistics for all of the variables of interest. We further conducted bivariate logistic regression analyses and multi-collinearity tests, to verify which variables to include in our model. Our final marginal model consisted of a logistic regression using generalized estimating equations (GEE) with institutional delivery as the outcome using the community capability score and demographic measures as explanatory variables [29]. The GEE model allows for non-independence in responses to produce a population-averaged or marginal model. Therefore, the marginal model allows the estimation of the odds of institutional deliveries as averaged over the entire sampled population. We further ran a series of mixed effects models, using the generalized linear latent and mixed models (GLLAMM) to identify the contribution of community level effects [30]. The GLLAMM method helped us to account for the intra-class correlation (ICC) between multiple observations within the same geographic areas. The mixed effects model we estimated with GLLAMM allows us a deeper exploration and quantification of the within sub-national unit (cluster) variability. Thus, it allows us to investigate the cluster-specific odds of institutional deliveries. Our final mixed effects model contained two levels, which assumed that households were nested within sub-national units. Because our data did not contain sufficient sample size at the village level, the sub-national unit was selected to represent the union level in Bangladesh; the sub-county level in Uganda; and the gram-panchayat in India. In addition to producing the odds ratio of interest and their respective confidence intervals, we also conducted an analysis of the various variance estimators. For the mixed effects model we calculated the intra-class correlation coefficient, which is a measure of the proportion of the total variation attributable to variation between clusters. We also explored the goodness of fit of these models.

## Results

Our final sample for analysis included 2775 women, of which 1238 were from Bangladesh, 1999 from India, and 338 from Uganda.

### Sample characteristics

In the pooled dataset, mothers’ average age was 27.22 years. The mother’s average age in our sample was similar across the three countries, though Indian mothers tended to be a bit younger than Bangladeshi and Ugandan mothers (24.97, 29.56, and 26.68, respectively), due to the fact that half of all mothers in the Indian sample were between 15 and 24 years old. About a quarter of all women in our sample had no formal education (24.9 %). More women in Uganda and India had primary education (72 and 53 %, respectively), than in Bangladesh (32 %). However, a larger proportion of women from the Bangladesh sample had lower secondary (26 %) or higher secondary (18 %) education than in the other two countries. Across the three countries, less than 1 % of the entire sample had university education.

Overall, women in the sample carried about 2.7 births to a viable gestational age. The highest parity was found amongst the Ugandan women (3.8), followed by the Bangladeshi women (3.25), and the Indian women (1.8). Across countries, most women across had three to five children (57 %). One fifth (21 %) of the sampled Bangladeshi women and one third (35 %) of the sampled Ugandan women had five or more children. Antenatal care (ANC) visits were common across the sample, with about one third (29 %) of all sampled women having attended one or two ANC sessions and almost two thirds (70 %) having attended three or more ANC sessions. In India and Uganda, most women attended three or more ANC sessions (86 and 78 %, respectively). In terms of the key maternal health outcomes of interest, a little over a third (35 %) of the sampled women had delivered in a health facility. Within our sample, Ugandan women most frequently delivered in an institution (71 %). Under half of the sampled Indian women delivered in a health facility (47 %). In Bangladesh, just 13 % of the women sampled delivered in a health facility. Further details about the sample characteristics are displayed in Table 3.

**Table 3:** Sample description of key variables in each country

Variables	Bangladesh (*n* = 1238)	India (*n* = 1199)	Uganda (*n* = 338)	Pooled estimates (*n* = 2775)
Community capability score (SD)	75.47 (15.02)	47.95 (17.45)	61.33 (15.89)	61.86 (20.71)
Mother’s age (%)				
15–24 years old	354 (28.59)	604 (50.38)	155 (45.86)	1113 (40.11)
25–34 years old	593 (47.90)	536 (44.70)	132 (39.05)	1261 (45.44)
35–49 years old	291 (23.51)	59 (4.92)	51 (15.09)	401 (14.45)
Mother’s mean age (SD)	29.56 (7.38)	24.97 (4.61)	26.68 (6.36)	27.22 (6.56)
Mother’s education (%)				
No formal education	278 (22.46)	385 (32.11)	28 (8.28)	691 (24.90)
Primary education	402 (32.47)	636 (53.04)	244 (72.19)	1282 (46.20)
Lower secondary education	321 (25.93)	130 (10.84)	64 (18.93)	515 (18.56)
Higher secondary education	226 (18.26)	36 (3.00)	1 (0.30)	263 (9.48))
University	11 (0.89)	12 (1.00)	1 (0.30)	24 (0.86)
Parity (%)				
One	232 (18.74)	513 (42.79)	64 (18.93)	809 (29.15)
Two-Four	742 (59.94)	686 (57.21)	156 (46.15)	1584 (57.08)
Five+	264 (21.32)	0 (0)	118 (34.91)	382 (13.77)
Parity mean (SD)	3.25 (1.98)	1.82 (0.88)	3.78 (2.41)	2.70 (1.84)
ANC number (%)				
None	0 (0)	17 (1.42)	1 (0.30)	18 (0.71)
One or two	513 (51.61)	148 (12.34)	72 (21.62)	733 (29.02)
Three or more	481 (48.39)	1034 (86.24)	260 (78.08)	1775 (70.27)
Institutional delivery (%)	172 (13.89)	562 (46.87)	241 (71.30)	975 (35.14)

### Community capabilities score

**Fig. 1:**
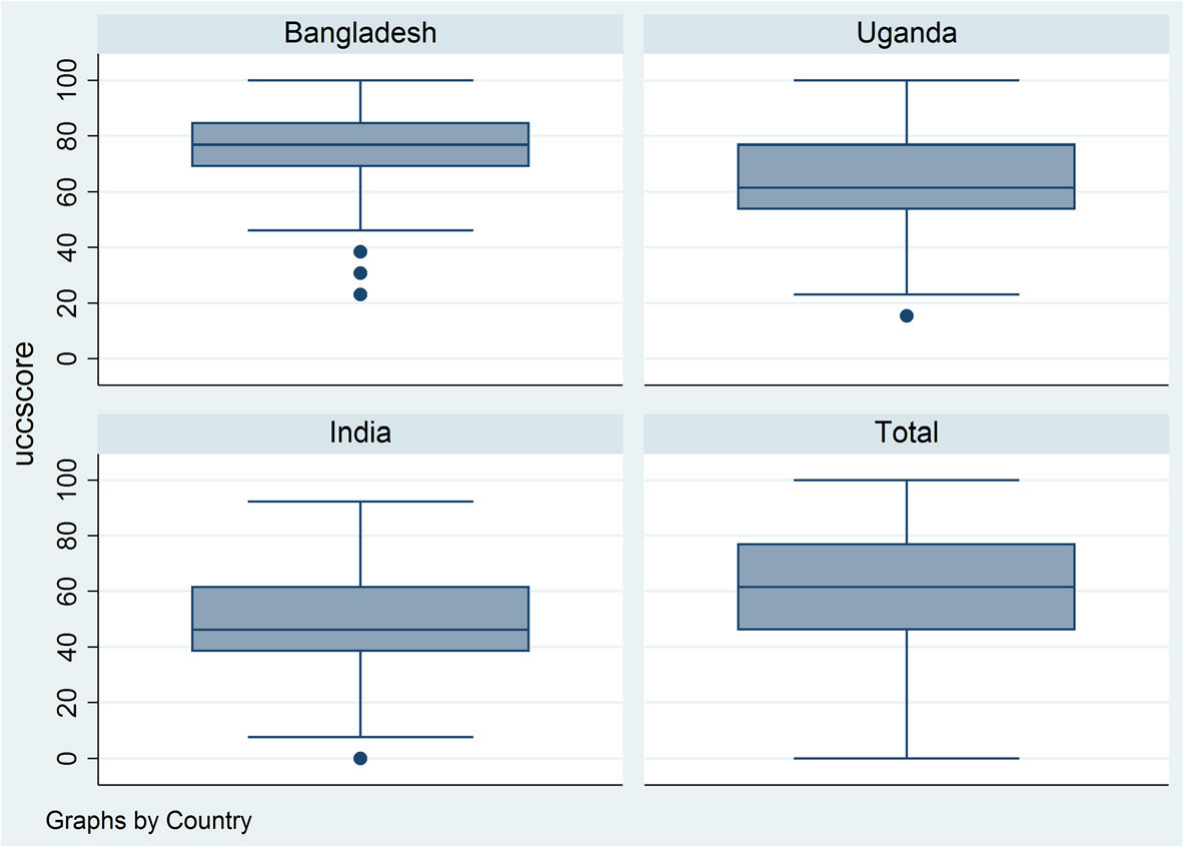
Mean community capability score in each country and in the pooled sample

**Fig. 2:**
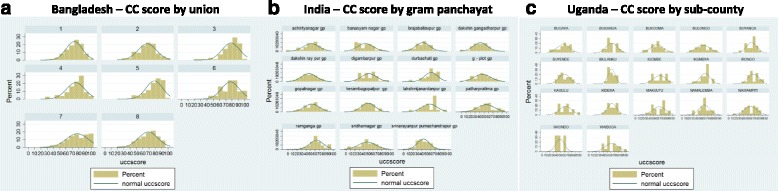
Distribution of community capability scores of households within each sub-national unit

The distribution of community capability scores across subnational units are illustrated Fig. 1 on a scale of 0 to 100. For the overall sample, the mean community capability score was 62, meaning that an average sub-national unit in our sample had access to 62 %, or about 8, of the 13 community capability items we explored (e.g. had access to village assets, had high community coherence questions, and had community organization participation). The highest community capability score were observed in Bangladesh, and the lowest ones in India. In Bangladesh, the average union reported had access to 75 % or between 9 and 10 of the 13 community capability items. In Uganda, the average sub-county had access to 61 % of community capability items. Finally, in India, the average gram panchayat had access to 48 % or roughly 6 of the 13 community capability items. A closer look at within country variation in community capability score, identifies some heterogeneity, as illustrated by the distribution of household scores of community capability within each subnational unit (Fig. 2).

### Individual and group determinants of institutional delivery

Table 4 summarizes the multivariate analysis results. From the mixed effects model, we discovered that, based on our sample, 5 % of the total variance is due to differences between sub-national units (i.e. clusters). This value is likely low because the country of origin and the individual-level predictors have the strongest effect.

**Table 4:** Multivariate logistic regression by characteristics of sample and factors associated with Institutional delivery

Independent variable	GEE regression	Mixed model
	Odds ratio (95 % CI)	Odds ratio (95 % CI)
Community capability score	1.0075 (1.0015, 1.0135)*	1.0077 (1.0018, 1.0136)**
Mother’s age [15–24 years old]		
25–34 years old	1.056 (0.8711, 1.2801)	1.0534 (0.8419, 1.3179)
35–49 years old	0.9844 (0.7202, 1.3457)	0.9859 (0.6899, 1.4088)
Mother’s education [No education]		
Primary education	1.4604 (1.1386, 1.8730)**	1.4952 (1.1656, 1.9180)**
Lower secondary education	1.6055 (1.2087, 2.1326)***	1.6573 (1.1927, 2.3029)***
Higher secondary education	3.5984 (2.4362, 5.3151)***	3.8216 (2.5482, 5.7314)***
University +	9.6071 (3.0953, 29.8179)***	10.6917 (3.4690, 32.9523)***
Parity [Two-Four children]		
One child	2.6155 (2.0865, 3.2787)***	2.7125 (2.1625, 3.4024)***
Five+ children	1.4031 (0.9755, 2.0182)	1.4260 (0.9700, 2.0962)
ANC number [None]		
One or two	2.8610 (0.9785, 8.3653)	3.8475 (0.4873, 30.3785)
Three or more	5.7897 (2.0831, 16.0910)***	8.0735 (1.0333, 63.0804)*
Country [Bangladesh]		
India	5.2516 (3.0000, 9.1920)***	5.4411 (3.2834, 9.0166)***
Uganda	15.0519 (8.8651, 25.5564)***	16.2267 (9.6068, 27.4082)***
Log-likelihood		−1331.8008
Variance of random effects (SE)		0.1984 (0.0771)
Intra-class correlation coefficient		0.0569

Notes: * <0.05; ** < 0.01 and *** <0.001

In both models, the effects of the individual and group variables on a woman’s odds of institutional delivery were similar. For example, the models revealed that maternal age was not a significant determinant of whether or not a mother would deliver at an institution, either at the population level or for women in a particular sub-national unit. Maternal education played a significant role in both cases. For example, the odds of institutional delivery were about 1.5 times greater for women who had completed primary education, compared to those who had no education at all, holding all other variables constant. A similar effect size was identified when analyzed at the sub-national level in the mixed effects model. The effect was much higher for women with higher secondary or university-level education. Parity was another significant determinant at both the overall and the sub-national unit level. The odds of institutional delivery were more than two and a half times higher for among women with one child, as compared to women with two to four children. Similarly, the odds of institutional delivery were almost one and a half times higher for women with five or more children than for women with one child, though this result was not significant in either of our models. Antenatal care was also an important determinant. The odds of institutional deliveries were significantly greater for women who had three or more antenatal care visits, compared to women who had none. The mixed model revealed a similar finding. Finally, the country of origin had a strong effect on the odds of institutional deliveries. Women in India and Uganda had much higher odds of delivering in a facility than women in Bangladesh. This finding held when comparing women in the same sub-national unit, holding all variables constant. This strong effect coincides with our sample characteristics (i.e. proportion delivering in an institution in each country), as well as with overall trends in institutional deliveries based on national-level maternal health surveys, as listed in the background section.

We found that community capability played a significant role in the odds of women delivering at facilities. The odds of institutional deliveries increased by 0.75 % for every unit increase in the community capability score. Based on our calculations, every one of the 13 capabilities included in the community capability score calculations corresponded to about 7.7 percentage points in our community capability score. Therefore, for every additional capability that the community obtained, there could be a 6 % increase in a woman’s odds for institutional delivery. An illustrative example might suggest that if a community that does not currently have a reliable water supply obtains it, the benefits of this community capability could potentially contribute to an increase (of up to 6 %) in the odds of women in that particular community delivering in a facility.

## Discussion

In this paper, we described the development of a measure of community capabilities, based on data available from Bangladesh, India, and Uganda. We then examined the role of community capability as a determinant of institutional deliveries, in these three contexts. We found that community capability was a significant and meaningful determinant in our analysis, although individual-level characteristics – such as maternal education, parity, ante-natal care visits, had the strongest effect on the odds of institutional deliveries. This is perhaps intuitive as the decision on where to deliver takes place in the home, and is influenced by the community context. To the best of our knowledge, in addition to Underwood’s study in Zambia, which found that community capacity had a significant effect on women’s contraceptive use, HIV testing, and children’s bed net use [19] and Brazier’s study that explored the association between community capacities for health promotion and the utilization of maternal health services [31], our team’s analysis represents one of the few attempts to document the role of community capability as a determinant of maternal health outcomes in a developing country context. This type of analysis can have many extensions, as other health outcomes, such as child health, would be worthwhile exploring further.

Our study conclusions, however, should be interpreted in the context of some limitations that were beyond our control. For example, each country team employed a different sampling technique and overall sample sizes were relatively small. Furthermore, because we wanted to focus on a cross-country analysis, we could not include variables that were not part of all country data sets. For example, while previous research has highlighted the role of other determinants of institutional deliveries, such as socio-economic status measures, fathers’ characteristics, other health systems barriers (e.g. distance to facility), we could not include these in our analyses as they were not consistently available in all data sets. Finally, our survey analysis did not include weighting to account for the various sample sizes in the three sites we analyzed. Each country used a different sampling technique, making it challenging to develop a weighing strategy for the pooled dataset, and our analysis focused on identifying associations rather than prevalence levels, so there is not a clear need to include sample weighting.

With respect to the community capability score, our calculation was simple, to ensure ease of interpretation. However, our approach assumed that all community capability had similar importance and weight, though the factor analysis suggested there were three main factors. In practice, some variables or factors may be more important in one context than another, and some may be more relevant to maternal health than others. Because we had to reduce items to include only those that were available across all countries, it is also possible that we omitted critical components that could have better explained the linkage with maternal health. Doing the analysis with all the components that are available for each country, as well as with the inclusion of determinants specific to a particular country would be very important and each country team may pursue this further analysis in future papers. For example, in this supplement, colleagues from the India team publish their findings on the role of community cohesion and membership in community organizations on child nutrition in disadvantaged communities [32]. Finally, although we had set out to calculate score at the community level, we did not have an adequate sample in all three countries to calculate the community capability score at the village level, with the sub-national unit providing a wider boundary for defining the community.

### Implications for future research

Our analysis raises further questions about how to refine our measurement of community capability and how to strengthen community capability to stimulate impacts on health. In this article, we have developed a simple community capability score. Developing measures that can be used to assess community capability across contexts is important. Therefore, future research should prioritize multi-country studies that use comparable instruments and use similar sampling strategies, so as to facilitate cross-country analyses of community capability and their role vis-à-vis health indicators. Furthermore, future research could examine how to better define community capability at the village or other locally-defined community level, so as to arrive as close as possible to measuring community capability at the true community-level, which might not always correspond to geographical boundaries. In addition to being tested across countries, the role of community capability in health could be further investigated within a particular country, perhaps comparing different regions (e.g. urban vs. rural). Larger samples in any of these analyses would help to better identify the cluster-specific contribution or effect. In addition, other ways of calculating the community capability score should be explored. For example, using all community capability items within a country could lead to more locally relevant scales and sub-scales. Finally, other variables, such as income, marital status, and distance to the nearest health facility, should be included in future analyses, as these have been shown already to be important determinants for access to maternal and other health services.

### Implications for program implementation and policy

Our analysis has important implications for program implementation and policy. The findings that, in addition to individual-level factors, the community-level or group-level factors also have an impact on whether or not a woman is able to deliver in an institution, points to a need for greater investment and exploration into how to strengthen community capability, as well as community participation in health systems interventions [33]. The context in which a woman resides should be taken into account when designing programs and interventions to support institutional deliveries [34]. Context-specific village or community-level interventions, such as the engagement and strengthening of existing and new community structures, as well as strengthening community-based service delivery and equity in access, are required to further support local health systems strengthening and adequate levels of community support for women to deliver safely in health facilities [35, 36]. Greater consideration of both the individual and community-level factors that influence access to health and overall well-being would also contribute to moving the entire global health community closer to the vision of supporting people-centered approaches for supporting resilience within health systems and communities.

## Conclusions

Community capability was a significant and meaningful determinant of institutional deliveries. The odds of institutional deliveries increased by 0.75 % for every unit increase in the community capability score. Based on our calculations, for every additional capability that the community obtained, there could be a 6 % increase in a woman’s odds for institutional delivery. While individual-level characteristics, such as maternal education, parity, ante-natal care visits, are strong determinants of whether a woman delivered in an institution, community capability should be taken into account when designing programs and interventions to support institutional deliveries. Consideration of individual factors and the capabilities of the communities in which people live would contribute to the vision of supporting people-centered approaches to health.

## Additional file

Additional file 1: Future Health Systems Research Consortium Unlocking Community Capabilities - Household Survey Measures. 2011, Johns Hopkins University School of Public Health - Future Health Systems Consortium: Baltimore, MD. (DOCX 20 kb)

## Abbreviations

ANC: Antenatal care
CCI: Community Capabilities Index
CHDSS: Chakaria Health and Demographic Surveillance System
FHS: Future Health Systems
GEE: Generalized estimating equations
GLLAMM: Generalized linear latent and mixed models
ICC: Intra-class correlation
icddr,b: The International Centre for Diarrhoeal Disease Research, Bangladesh
IIHMR: Indian Institute of Health Management Research
MakSPH-HDREC: Makerere University School of Public Health Higher Degrees Research and Ethics Committee
MANEST: MAternal and NEwborn care practices STudy
PPS: Probability proportionate to size
SASCAT: Shortened Adapted Social Capital Assessment Tool
