# Supplementary material for: What is the role of community capabilities for maternal health? An exploration of community capabilities as determinants to institutional deliveries in Bangladesh, India, and Uganda
Source: BMC Health Serv Res. 2016 Nov 15;16(Suppl 7):61–71. doi: 10.1186/s12913-016-1861-0 (PMC5123293; doi:10.1186/s12913-016-1861-0)
Supplement: Additional file 1: — Future Health Systems Research Consortium Unlocking Community Capabilities - Household Survey Measures. 2011, Johns Hopkins University School of Public Health - Future Health Systems Consortium: Baltimore, MD. (DOCX 20 kb) [file 12913_2016_1861_MOESM1_ESM.docx]

**Future Health Systems Research Consortium**

**Unlocking Community Capabilities**

**Household Survey Measures**

The purpose of this note is to provide guidance for a set of quantitative measurements to be used in research across Future Health Systems to assess community capability. The questions are intended to be used in household surveys by the research teams, but the same questions can be asked in exit surveys or other surveys conducted by the teams. They can also complement qualitative research or be integrated into mixed-methods research on community capabilities. The concepts of community capabilities, the agency and opportunity structures to access community resources, and the roles of power and relationships in communities are multi-dimensional and highly contextualized. In order to aid comparison and understanding, the questions are organized under conceptual domain of community capabilities, so that teams may select what is most relevant to their research. Teams may also want to add their own questions that are pertinent to their situation.

**Questions selection criteria.** The questions are selected to represent the following criteria:

- Represent a measure of one or more domains of community capability
- Limited number of questions, and a limited variety of scale responses to promote simplicity and not overburden respondents
- Previously published so that comparisons can be made with other research
- Close-ended so that coding and analysis can be more uniform across sites, and to ease translation
- Can be used to construct a scale demonstrating more or less quantity of a dimension of community capability

**Cross-Country Use**. The products include the questionnaire modules and robust scales that measure community capability. These can be used by the research teams to measure different dimensions of community capabilities at community and household levels, and used in analysis as outcome measures or as important independent variables.

**Thematic Team Use.** Many questionnaires have been used to assess different types of community capabilities, but formal tests of reliability and validity of the instruments have not been published, particularly across different environments. The thematic team proposes to undertake reliability and validity analysis based on baseline or early household surveys that are being used across FHS, and to develop more formal scales of community capabilities and household and individual access to community capabilities. Formal reliability analysis involves the reproducibility of the results of an instrument (formally defined as the ratio of subject variability to subject variability plus measurement error). This analysis will include measures taken from repeating the questionnaire by different raters (inter-rater reliability), repeating the same questionnaire in the same subject at different times (test-retest reliability), and assessing the internal correlations of items in a scale. Validity analysis will examine the degree to which the scales measure what they are intended to measure. This analysis will include assessing the degree to which the measurements address the phenomenon of community capability (content validity), the degree to which the measurements correspond to the theoretical domains (construct validity), and the degree to which the measurements are associated with other externally verifiable assessments of community capability (criterion validity).

**Domains and Questions.** The following table identifies the main domains to be covered in the questionnaire, a working description of the domain, and the module(s) that correspond to each domain.

**Domains and Question Modules**

| **Domain** | **Definition** | **Question Module** |
| --- | --- | --- |
| 1. Community Assets |  |  |
| 1a. Physical and Organizational | The physical and organizational resources of a community to which members of the community should have access, and the ability of communities to mobilize resources for collective use. | A & B |
| 1b. Information | The degree to which members of a community have similar and sufficient knowledge about issues and programs, and access to information sources | A |
| 1c. Institutional | Institutional arrangements that enable a community to enhance its capacity and maintain traditions, norms, and institutions, including the accountability of local organizations | A, B, C, D, E |
| 2. Group Participation | The community’s capacity to engage its members in collective action, and the degree to which members are active in group functions | B |
| 3. Voice in Community | The ability for community members to express a desire concerning leadership of the community or use of community resources, often through political processes | C |
| 4. Collective Efficacy | The group’s shared ability to undertake action to attain their goals, solve problems, and produce results that have an impact on their community. | D |
| 5. Collective Critical Thinking | The ability of the community to consider alternative ways of thinking and reflect on assumptions underlying their action and results of past actions (Note: this is also a particular type of institutional asset) | D |
| 6. Social Cohesion | The forces that act on members of a community to remain in and actively contribute to the community. Cohesiveness measures the degree to which members want to be part of a group and are loyal and united in pursuit of group goals. | E |
| 7. Community Leadership | The ability of individuals within the community to bring about change in and by the community, including the ability to represent different group interests across the community, and to exercise power and decision-making collectively | E |
